# Multiple Myeloma Presenting as a Fistulous Rectal Mass Eroding Into the Retroperitoneum

**DOI:** 10.14309/crj.0000000000000621

**Published:** 2021-07-02

**Authors:** Taseen Syed, Thaer Abdelfattah, Patricija Zot, Ravi Vachhani

**Affiliations:** 1 Department of Gastroenterology, Hepatology, and Nutrition, Virginia Commonwealth University, Richmond, VA; 2 Department of Gastroenterology, McGuire VA Medical Center, Richmond, VA; 3 Department of Pathology, Virginia Commonwealth University, Richmond, VA

## CASE REPORT

**Figure 1.**
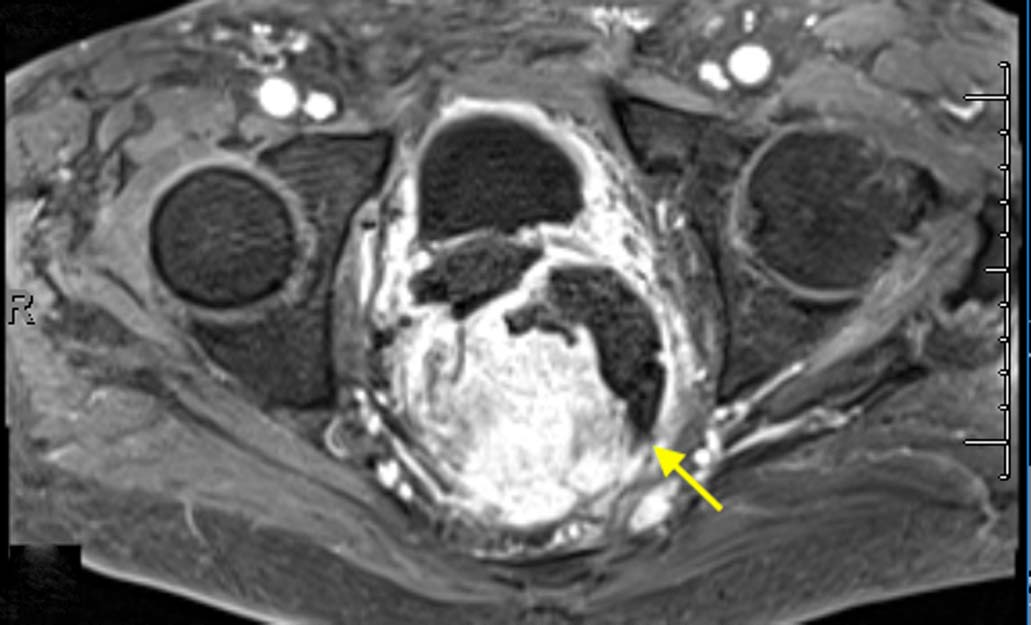
Abdominal magnetic resonance showing a large pelvic mass with circumferential involvement of the rectum and evidence of contained perforation.

**Figure 2.**
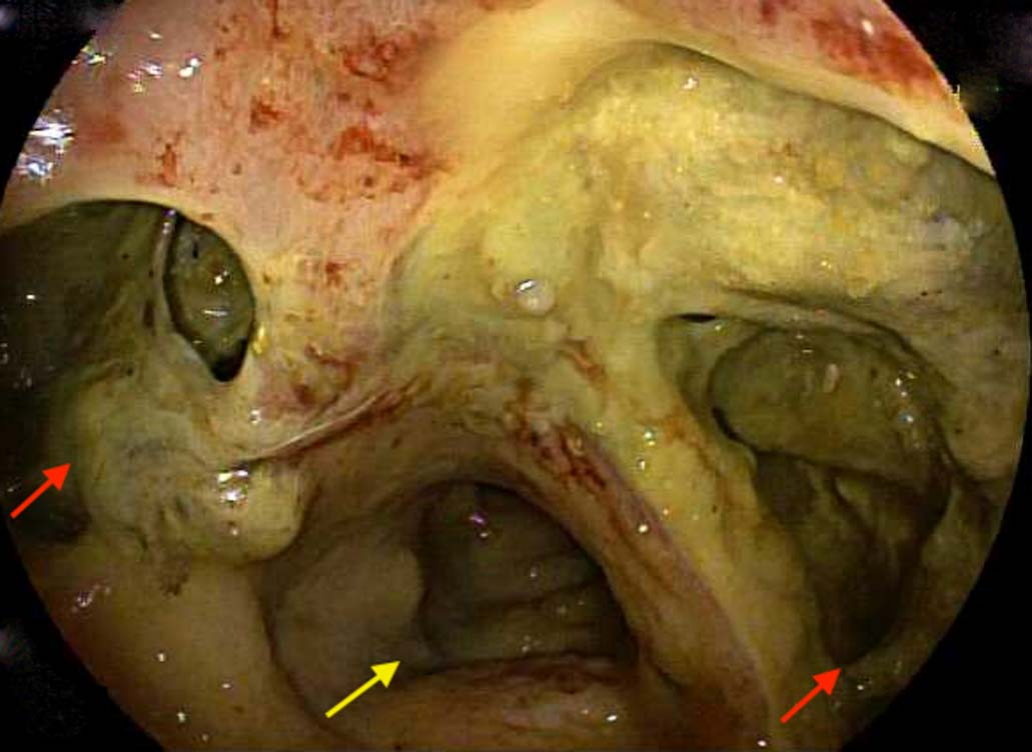
Sigmoidoscopy confirmed a large ulcerated mass (red arrows) eroding into the retroperitoneum and perirectal space. Yellow arrows show the lumen.

**Figure 3.**
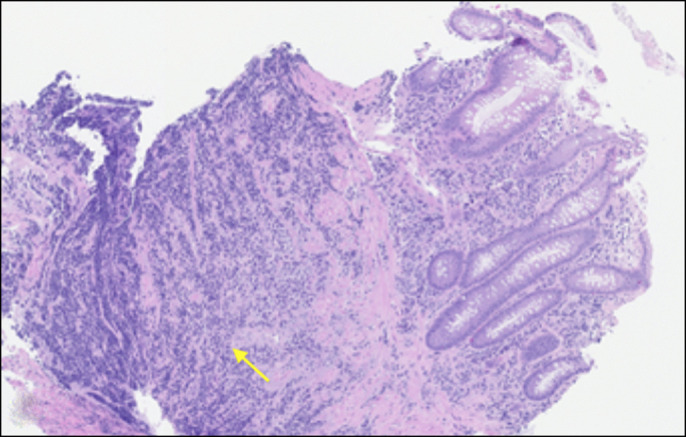
Biopsy showing a diffuse infiltrate of plasma cells with marked nuclear atypia within the lamina propria and submucosa that was consistent with plasma cell neoplasm.

An 84-year-old African American man with a medical history of prostate cancer presented with subjective fevers and abdominal pain. He denied nonsteroidal anti-inflammatory drug use or tobacco use. Surgical history included prostatectomy without radiation therapy. Physical examination was unremarkable other than generalized abdominal pain. Laboratory studies were within normal limits as well, including a complete blood count and comprehensive metabolic panel. Magnetic resonance imaging of the abdomen showed a large pelvic mass with circumferential involvement of the rectum with evidence of contained perforation (Figure 1). Subsequently, a flexible sigmoidoscopy confirmed a large ulcerated mass eroding into the retroperitoneum and perirectal space shown by red arrows (Figure 2). Biopsies showed diffuse infiltrate of plasma cells with marked nuclear atypia within the lamina propria and submucosa that was consistent with plasma cell neoplasm (Figure 3).

The patient was referred to a multidisciplinary oncology team for further evaluation and management. Extramedullary multiple myeloma involving the colon is a rare presentation with rectal involvement as an eroding mass along with fistulous tracts not reported before.^1^ Although gastrointestinal plasma cell neoplasms are rare, they must be considered in patients presenting with gastrointestinal symptoms. The suspicion should increase in patients with a previous diagnosis of multiple myeloma.^2^

## DISCLOSURES

Author contributions: T. Syed and T. Abdelfattah wrote the manuscript and reviewed the literature. P. Zot edited the manuscript. R. Vachhani approved the final manuscript and is the article guarantor.

Financial disclosure: None to report.

Informed consent was obtained for this case report.

## References

[1] Kakati BR Krishna K Krishna SG Sharma SG Sanathkumar N Rego RF. Extensive extramedullary disease involving the colon in multiple myeloma: A case report and review of literature. J Gastrointest Cancer. 2012;43(2):379–81. 20703830 10.1007/s12029-010-9199-z

[2] George SM Aljufairi EA Chandran N Almahari SAI. Plasmacytoma as a mimicker of colonic carcinoma in an elderly man. Case Rep Pathol. 2017;2017:4846018. 28503336 10.1155/2017/4846018 PMC5414488

